# Neurological Assessment and Its Relationship to CSF Biomarkers in Amateur Boxers

**DOI:** 10.1371/journal.pone.0099870

**Published:** 2014-06-18

**Authors:** Sanna Neselius, Helena Brisby, Jan Marcusson, Henrik Zetterberg, Kaj Blennow, Thomas Karlsson

**Affiliations:** 1 Department of Orthopaedics, Sahlgrenska University Hospital, Gothenburg, Sweden; 2 Institute for Clinical Sciences, the Sahlgrenska Academy at the University of Gothenburg, Gothenburg, Sweden; 3 Geriatric Section, University Hospital in Linköping, Linköping, Sweden; 4 Institution of Clinical and Experimental Medicine, Linköping University, Linköping, Sweden; 5 Clinical Neurochemistry Laboratory, Sahlgrenska University Hospital, Mölndal, Sweden; 6 Institute of Neuroscience and Physiology, Department of Psychiatry and Neurochemistry, the Sahlgrenska Academy at the University of Gothenburg, Gothenburg, Sweden; 7 UCL Institute of Neurology, Queen Square, London, United Kingdom; 8 Disability Research, Department of Behavioral Sciences and Learning, Linköping University, Linköping, Sweden; 9 Linnaeus Centre HEAD, Linköping University, Linköping, Sweden; University Of São Paulo, Brazil

## Abstract

**Background:**

Mild traumatic brain injury (TBI) or concussion is common in many sports. Today, neuropsychological evaluation is recommended in the monitoring of a concussion and in return-to-play considerations. To investigate the sensitivity of neuropsychological assessment, we tested amateur boxers post bout and compared with controls. Further the relationship between neuropsychological test results and brain injury biomarkers in the cerebrospinal fluid (CSF) were investigated.

**Method:**

Thirty amateur boxers on high elite level with a minimum of 45 bouts and 25 non-boxing matched controls were included. Memory tests (Rey Osterrieth Complex Figure, Listening Span, Digit Span, Controlled Word Association Test, and computerized testing of episodic memory), tests of processing speed and executive functions (Trail Making, Reaction Time, and Finger Tapping) were performed and related to previously published CSF biomarker results for the axonal injury marker neurofilament light (NFL).

**Results:**

The neurological assessment showed no significant differences between boxers and controls, although elevated CSF NFL, as a sign of axonal injury, was detected in about 80% of the boxers 1–6 days post bout. The investigation of the relationship between neuropsychological evaluation and CSF NFL concentrations revealed that boxers with persisting NFL concentration elevation after at least 14 days resting time post bout, had a significantly poorer performance on Trail Making A (p = 0.041) and Simple Reaction Time (p = 0.042) compared to other boxers.

**Conclusion:**

This is the first study showing traumatic axonal brain injury can be present without measureable cognitive impairment. The repetitive, subconcussive head trauma in amateur boxing causes axonal injury that can be detected with analysis of CSF NFL, but is not sufficient to produce impairment in memory tests, tests of processing speed, or executive functions. The association of prolonged CSF NFL increase in boxers with impairment of processing speed is an interesting observation, which needs to be verified in larger studies.

## Introduction

Mild traumatic brain injury (TBI) or concussion is defined as a complex pathophysiologic process affecting the brain, induced by traumatic biomechanical forces with or without loss of consciousness [1]. They represent 80–90% of all traumatic brain injuries [1] and account for between 5.8–8.9% of all athlete injuries among United States high school and collegiate athletes [2]. It is also worth pointing out that most athletes experiencing a concussion, up to 70%, do not seek medical attention [3]. Since concussion is now recognized as an increasing and serious health problem in many sports, it has gained increased attention among medical professionals, scientists, sport organizations as well as athletes.

A TBI causes axonal and glial damage, which disturbs the cerebral physiology, and makes the brain more vulnerable for additional concussions. The primary neuropathology of mild TBI is a diffuse axonal injury (DAI) [4], [5]. It is caused by shearing of fragile axons, due to acceleration and deceleration forces during the trauma [6]. The DAI inflicted by a mild TBI, causes predominantly cell dysfunction, rather than cell death [7]. A boxer can receive a mild TBI due to a knockout punch. The knockout frequency in modern amateur boxing is very low, in average 0.7% [8], however, it is known that also the repetitive subconcussive trauma in boxing causes axonal injury [9]–[12].

### Neuropsychological Evaluation of Mild Traumatic Brain Injury

Neuropsychological evaluation has been advocated as the most sensitive tool for early detection of central nervous system pathology and numerous studies have shown that both traditional and computerized neuropsychological tests (emphasizing mental processing efficiency and speed) are sensitive for the acute cognitive impairment after a mild TBI up to 10 days post injury [13]–[16]. Also in studies where patients with mild TBI have been followed for longer time periods have concluded that neuropsychological assessment in mild TBI are of clinical value and contributes with significant information in the evaluation, in combination with symptom and physical assessment [17], [18]. In a meta-analysis of neuropsychological outcome after a single mild TBI, the largest impairment was seen in verbal, visual and working memory (attention, concentration, processing speed) up to 30 days post trauma [17]. However, the largest effect was observed during the first seven days and no neuropsychological changes were detectable after three months [17]. In a study on 698 jockeys decrements in high-level executive/attentional functioning after multiple concussions, persisting more than three months post trauma, were seen [18]. Younger athletes were here found to be more vulnerable to concussions than adults.

For amateur boxing there is a recently published study showing cognitive impairment, after self-reported concussions (knockouts), when compared to controls [19]. Here, cognitive recovery occurred within a week. In this study the computerized test Automated Neuropsychological Assessment Metrics (ANAM) [20] was administered and the study objects in the boxer group were tested at baseline and post injury and thereafter compared to a group of baseline tested boxers without concussion. However, to our knowledge, no neuropsychological assessment has been able to show any pathology caused by the repetitive subconcussive trauma in amateur boxing [21]–[23], indicating that there are no cognitive tests that are sensitive enough to detect small axonal injuries.

### Neuropsychological Tests used in the Diagnosis and Monitoring of TBI

#### Episodic memory

**The Rey-Osterrieth Complex Figure (ROCF) Test** is commonly used to test episodic memory and visuospatial skills [24]. It correlates with the Trail Making test and Wechsler Adult Intelligence Scale – Revised (WAIS-R) [25]. A study comparing subjects with traumatic brain injury (TBI) and patients with Alzheimer’s disease (AD) showed that the TBI subjects did not have any dysfunctions in copying the figure, only with recall, but the AD subjects had dysfunctions both with copying and recall [26]. There are also computerized possibilities, for example testing made by a model from Tulving [27], to evaluate episodic memory.

Episodic memory after severe TBI has been shown to be impaired initially [28], but no long-term consequences have been seen after mild to moderate traumatic brain injury [29].

#### Language and semantic memory

**Vocabulary intervention** is part of the WAIS-R and related to level of education and is critical for studies where educational background may interfere with neuropsychological results [30].

**Controlled Oral Word Association Test** (COWAT) is a part of the Multilingual Aphasia Examination and provides a measure of word retrieval and word fluency [31], [32]. A study on 96 athletes suffering from a sport-related concussion did not reveal significant difference with controls [33], but in mild cognitive impairment (preclinical stage of AD), impairment was seen compared to controls [34].

#### Working memory

**Listening span Test** has been used extensively in our laboratories [35] and measures complex, executive aspects of working memory that are related to short-term memory capacity [36], [37]. It is impaired in preclinical stages of AD [38] but seems not to be affected by sport-related concussions [39]. **Digit Span Test** is part of the Wechsler Adult Intelligence Scale – Revised (WAIS-R) and addresses short-term auditory memory that is part of the working memory [30]. It can be used to monitor less effortful attention skills, in contrast to the Listening Span task, that involves effortful working memory and attention skills. Typically, performance on the Digit Span task is relatively insensitive to effects of mild TBI [40]. However, it ascertains that participants master necessary attention skills in order to allow meaningful interpretation of other neuropsychological data.

#### Processing speed and executive functions

**Trail Making Test** is used for assessing processing speed, attention and executive functioning [41]. There is ample evidence that it can detect brain damage and predict long-term outcomes after traumatic brain injury [42]–[44]. **Reaction time Test** is sensitive to the effects of mild TBI [45].

Previous studies have reported that **Finger tapping Test** is impaired after a mild TBI [46], [47] and that boxers show a reduced performance compared to controls [48].

### Evaluation of Biomarkers in Cerebrospinal Fluid and Blood in this Cohort of Boxers

We have previously shown that amateur boxing causes elevation of brain injury biomarkers in blood and CSF [9]–[11]. CSF Neurofilament Light Protein (NFL), a marker of axonal injury, was shown to be the most sensitive biomarker and to correlate with the amount of injury [9]. NFL levels were elevated in 77% of the boxers 1–6 days after a bout as a sign of mild TBI, when compared to matched controls. After a rest period of at least 14 days, the levels were still elevated in 46% of the boxers [9].

### Aim of the Study

The primary aim of the study was to investigate if neurological assessment can detect cognitive impairment caused by subconcussive trauma in amateur boxing and if possible deficits are related to specific cognitive domains. Secondly, as the first study, we sought to investigate the relationship between neuropsychological evaluation and CSF NFL, previously analyzed in the same cohort [9].

## Method

### Study Population

The study was designed as a prospective prognostic follow-up study. Thirty Olympic boxers competing at high national and/or international level were compared to 25 healthy, age-matched controls. All boxers had completed at least 45 bouts. This inclusion criterion was based on the regulation of the National Boxing Federation demanding an examination including magnetic resonance imaging (MRI), computer tomography (CT) or electroencephalography (EEG) every 50 bouts. The controls consisted of friends or relatives to the boxers, aiming to make certain the enrolment of controls with similar social background and education level similar to the boxers. Exclusion criteria included performance at elite level in sports where head trauma frequently occurs, such a soccer, ice hockey and martial arts. On the same cohort results from biomarker analysis in blood and CSF have been previously published [9]–[11].

### Ethical Approval

The regional ethical review board at Linköping Health University, Sweden approved the study. Written informed consent was obtained from all participants.

### Magnetic Resonance Imaging

Magnetic resonance imaging (MRI) of the brain was performed in all participants without any structural injuries (haemorrhages, subdural haematomas) or other major findings observed.

### Questionnaire Design

All participants filled in a questionnaire about medical history, medication, education, present occupation, information about previous concussions and quantification of alcohol and drug intake. Previous sports career was reported to identify those who had trained in sports with risk of TBI. The questionnaire included a 10-question symptom evaluation of head and neck injuries based on a previous study [49]. The number of symptoms that had worsened over the last 5–10 years was combined in a score. The boxers reported about their boxing career; fighting record, number of knock-out losses, number of Referee Stopping Contest losses due to several hard punches to Head (RSC-H), present weight class, duration of career, age at career start and age at first bout [49], [50]. Boxers gave an account for total amount of bouts the last week prior testing (1–3 bouts) and estimated these bouts as easy (1), intermediate (2) or tough (3). Three boxing experts independently (without knowledge of any results) graded the boxers considering head trauma during total boxing career, 1 to 5 (Type 1 is a boxer that has a low risk to receive blows to the head, according to boxing style, skills and the skills of the opponents. Type 5 is a boxer with high risk to receive repeated blows to the head). The total amount of bouts the last week before test A, the boxers own grading of the bouts and the mean of the expert grading were combined in a score named “Boxing Exposure”. The aim was to calculate the total mild TBI risk prior testing.

### Neurological Examination

All participants underwent a neurological examination by the same physician [51]. The neurological examination protocol included anamnestic questions about concussion symptoms, a general somatic status (general condition, examination of mouth and throat, heart, blood pressure, abdominal palpation, peripheral circulation and skin status) and a neurological status (GCS, orientation, alertness, speech function, cranial nerves 1–12, motor skills, balance testing, coordination, gate, sensibility testing and testing of reflexes).

### Neuropsychological Assessment

The cognitive testing was administered at 1–6 days after the last bout (in average 2 days after bout), prior to, but at the same day as CSF and blood tests (test A) [9]. The cognitive testing was performed in a quiet room without distraction at the university hospital at daytime. The same examiner administrated all the tests following a standardized procedure. The duration for the assessment was approximately 60 minutes. A blinded experienced neuropsychologist analyzed the results. Following parts in presented order constituted the test:

#### 1. Rey osterrieth complex figure test, part 1

Part one involved the untimed reproduction of a complex figure [24].

#### 2. Vocabulary test

The task involved the explanation of the meaning of words, ranging from common to less well-known items.

#### 3. Controlled Oral Word Association Test (COWAT)

Participants were asked to generate as many words as possible that begins with a given letter, (i.e. F, A OR S, excluding proper names, numbers or words with different tenses or endings). Sixty seconds was allowed for each letter. The dependent variable was the total number of correct words produced, minus any repetitions [24].

#### 4. Listening span test

The participants listened to a set of sentences, half of which were semantically correct and the other half incorrect. Participants were instructed to report whether each sentence was correct or not and to remember the last word in each sentence. This procedure was repeated for two to five sentences. After the sentences had been presented, the participants were asked to recall all the target words in correct order. The task was repeated five times at each level of difficulty [52].

#### 5. Rey osterrieth complex figure test, part 2

The second part of the ROCF was included after the Listening Span Test and involved the reproduction (i.e., delayed recall) of the complex figure 30 minutes after the first presentation.

#### 6. Computerized testing: episodic memory – part one

This test was computerized and constructed by a model from Tulving [27]. Two consecutive lists, one visual and one auditory list containing 36 words each were presented. The visual list was presented on a computer screen and the other list was presented auditory from computer. All words were presented once with interstimulus interval of 2.0 s. Presentation of the words was randomized. After the presentation of the two lists the participants were informed that the test would occur at a later point.

#### 7. Digit span

Participants were presented with series of numbers, starting with three (for example 3–4–8). There were two series in each level and in total seven levels. The task was to immediately repeat the numbers back. When doing this successfully, the participants were allowed to continue to next level.

#### 8. Trail making test, A and B

In trail A the participant was instructed to trace a line that connects circled numbers in consecutive order. In trail B the task was to trace a line by alternating circled numbers and circled letters in consecutive order (1–A–2–B–3–C, and so on) [53].

#### 9. Computerized testing: simple and complex reaction time

The test was computerized and constructed by a standardized model [54]. In our version of the task, participants were presented with one of two geometrical figures (a circle or a triangle). The size of the stimuli was approximately 80×80 mm. In the condition involving simple reaction time, participants were instructed to respond as quickly as possible whenever the circle appeared on the screen. Complex reaction time required participants to respond with right index finger to the circle and left index finger to the triangle. Each stimulus was presented for 100 msec. A randomly varying interstimulus interval (ISI) was used, ranging between 300 and 5000 msec. The measurement was based on 40 repetitions of each condition. Individual mean values for the simple and complex reaction time were calculated and the difference between complex and simple reaction time used in the further analysis of the results.

#### 10. Computerized testing: Finger Tapping

The task is a modified version of the Halstead-Reitan Finger Tapping (or Oscillation) Test [55], [56].

The participants were asked to keep their right hand palm down; fingers extended, and rest the index finger on a designated key (the space bar) on a computer keyboard. The participants were instructed to press the key as many times and as fast as possible until a brief pause was introduced. The entire session consisted of five consecutive trials of 15 seconds each with a 15 second rest in between trials. We used the pace ( =  mean number of finger taps across trials) in our further analyses.

#### 11. Computerized testing: episodic memory – part two

Following the part one of the Episodic Memory task, a self-paced, computerized, yes-no recognition test took place. In this test, the words from the two previous word lists were presented, together with distracting words. That is, half the target words were previously listed in the free recall task, half of the target words were uncontaminated by previous attempts at free recall.

Along with the presentation of each word, the participants were asked whether the word had occurred in the previous study lists. When an affirmative answer was given, the subject was also asked to decide if the recognition was accompanied by recollection. If a yes response was connected to an explicit recollection from the presentation earlier, the subject was asked to provide a remember response. The participants were informed that recollections could be associations that took place during the previous presentation, feelings or thoughts that linked the affirmative recognition decision to the previous presentation of the specific word. On the other hand, if the yes-response was not associated with a specific memory (i.e., the decision was based upon another criteria than explicit recollection, such as familiarity or ease of perceptual fluency), the subject was asked to provide a know response. Written instructions explaining the nature of the recollection classification task (including particular examples) were presented on the computer screen during the recognition test. In addition, the experimenter provided additional examples and explanations when the subject found the distinction between knowing and remembering difficult to use.

When the word recognition task was completed followed another word recognition task, also a self-paced computerized, yes-no recognition test. In this test, the participant was asked to discriminate and identify earlier presented audile words among earlier visual presented words and fifteen new distracters. Along with the presentation of each word, the participant was asked to decide whether the word had occurred in the previous study lists as audile stimuli. When an affirmative answer was given, the subject was also asked to decide if the recognition was accompanied by recollection. If a yes response was connected with an explicit recollection from the presentation earlier on, the subject was asked to provide a remember response. On the other hand, if the yes-response was not associated with a specific memory (i.e., the decision was based upon another criteria than explicit recollection, such as familiarity or ease of perceptual fluency), the subject was asked to provide a know response. Also in this task written instructions explaining the nature of the recollection classification task (including particular examples) were presented on the computer screen during the recognition test.

### Investigation of Relationship between Neurological Assessment and CSF NFL

Boxers with NFL concentrations elevated more than 2 SD above the control mean, were considered to have abnormal levels.

### Statistics

Statistical analysis was carried out with IBM SPSS Statistics 21.0. Comparisons between groups were performed using the non-parametric Mann-Whitney U-test, due to sample sizes and since some of variables had skewed distributed data, following an initial MANOVA (multivariate analysis of variance). When variables did not meet criteria for conventional MANOVA procedures, the initial MANOVA was carried out on ranked results [57].

## Results

### Questionnaire Design and Neurological Examination

The questionnaire about medical and social history and the 10- question survey were similar between boxers and controls [9]. None of the boxers suffered from loss of consciousness during their last bout before test A. Only one of the boxers reported concussion related symptoms after the bout (in this case headache) at the clinical examination, but the medical and neurological examination was normal in all subjects, GCS 15.

### Neuropsychological Assessment

Criteria for statistical significance was not reached with an initial MANOVA encompassing neuropsychological results, except for the tests Rey-Osterrieth Figure - part two and Auditory Know HR (F(14,40) = 1.04, p = 0.44). These two did not fulfill criteria for the use of conventional MANOVA. MANOVA of ranked data failed to disclose a statistically significant effect (L(2) = 0.11, p = 0.74). Univariate results are shown in Table 1.

**Table 1:** Results of the neuropsychological assessment for boxers vs. controls.

TEST	BOXERSN = 30Mean (range) SD	CONTROLSN = 25Mean (range) SD	P-valueBoxers vs. Controls
**ROCF** 1			
Copy (max 36)	33.6 (8.0–36) 6.6	35.3 (31–36) 1.2	0.78
Delay (max 36)	17.9 (1–31) 8.3	19.1 (6.5–34) 8.0	0.71
**VOCABULARY** 2 **(max 70)**	31.9 (16–60) 11.3	30.5 (15–46) 9.5	0.61
**COWAT** 3	36.9 (18–54) 10.7	37.6 (15–53) 9.2	0.91
**DIGIT SPAN** 4 **(max 14)**	7.1 (3–11) 2.0	6.5 (4–12) 2.0	0.13
**LISTENING SPAN** 5 **(max 38)**	14.3 (1–33) 7.6	10.6 (2–29) 6.6	**0.05**
**TRAILMAKING** 6			
Part A, (s)	29.9 (15–75) 13.0	29.2 (17–51) 8.9	0.68
Part B, (s)	80.5 (40–240) 39.6	81.3 (41–210) 34.3	0.61
**REACTION TIME** 7			
Simple (msec)	323.3 (233–951) 131.1	297.4 (229–652) 80.6	0.37
Difference (complex-simple, msec)	245.4 (−248–3088) 549.0	203.8 (49–506) 119.6	0.31
**FINGER TAPPING** 8			
Dominant Hand	52.2 (range) 10.8	52.8 (range) 16.0	0.94
Non-Dominant Hand	58.2 (range) 14.7	57.1 (range) 17.6	0.68
**EPISODIC MEMORY** 9			
Auditory – Remember HR	0.41 (0.04–0.86) 0.24	0.35 (0.04–0.77) 0.27	0.22
Auditory – Know HR	0.18 (0.04–0.68) 0.18	0.24 (0.04–0.77) 0.24	0.95
Visual – Remember HR	0.31 (0.04–0.77) 0.24	0.18 (0.04–0.68) 0.16	**0.02**
Visual – Know HR	0.16 (0.04–0.86) 0.16	0.15 (0.04–0.5.) 0.13	0.33

The boxers performed significantly better than the controls in the Listening Span and Visual – Remember HR tests (p = 0.05 and 0.02 respectively). No other significant differences were seen between the groups.

1 *ROCF: Rey Osterrieth Complex Figure Test.*

2 *Vocabulary: 35 words were presented and for each word 0,1 or 2 points were assigned.*

3 *COWAT (Controlled Word Association Test): Participants were asked to generate as many words as possible that begins with the given letters F, A and S. Sixty seconds were allowed for each letter. One point per word was received.*

4 *Digit Span: Participants were presented with series of numbers, starting with three (for example 3–4–8). There were two series in each level and in total seven levels. The task was to immediately repeat the numbers back. When doing this successfully, the participants were given a longer serie of numbers.*

5 *Listening Span: A mixed lists of digits and letters were read aloud to the participants and they were asked to recall this list in correct numeric and alphabetic order.*

6 *Trail Making: In trail A the participant is instructed to trace a line that connects circled numbers in consecutive order. In trail B the task is to trace a line by alternating circled numbers and circled letters in consecutive order. Time is measured in seconds(s).*

7 *Reaction time: a) Simple reaction time: Participants were instructed to respond as quickly as possible whenever the circle appeared on the screen. Complex reaction time: Required participants to respond with right hand to the circle and left hand to the triangle. Difference = Complex−Simple reaction time.*

8 *Finger Tapping. In total five trials with 15 s pauses between the trials. The participants were asked to press the space board with their index finger on the computer keyboard as many times as possible for 15 s, alternating dominate and non-dominant hand. The mean value was calculated.*

9 *Propotional values (range 0.04–0.96). There are 30 words respectively in the auditory and visual conditions. The proportion of correct recognition hit rates (HR) is presented.*

#### Episodic, language, semantic and working memory

The scoring in the Rey-Osterrieth Figure Test was made according to established criteria developed by David Loring [58]. Nor the Rey-Osterrieth Figure test or “the delay and episodic memory computer task” revealed any impairment in the boxers compared to controls. In one condition (Visual – Remember HR) the boxers presented with a larger proportion of ‘know’ responses (p = 0.02) than the control group (Table 1).

The test parameters Vocabulary and COWAT showed no differences between the groups (Table 1).

Digit Span and Listening Span were used to assess working memory. No differences were seen in Digit Span but the boxers performed better than the matched controls in the Listening Span Task (p = 0.049, Table 1).

#### Processing speed and executive functions

Trail Making, Reaction Time and Finger Tapping were used to evaluate processing speed and executive functions. No differences between the groups were detected in any of those tests.

#### Visuospatial ability: rey-osterrieth figure test, part one

Performance on ROCF was similar in boxers and controls (Table 1).

### Investigation of Relationship between Neuropsychological Assessment and CSF NFL

Criteria for statistical significance was not reached with an initial MANOVA encompassing neuropsychological results, except for the tests Trail Making B and Simple Reaction Time. (F (14) = 1.4, p = 0.26). These two did not fulfill criteria for the use of conventional MANOVA. MANOVA of ranked data indicated a statistically significant effect (L(2) = 5.4, p = 0.02). Univariate results are shown in Table 2.

**Table 2:** Investigation of relationship between neurological assessment and neurofilament light (NFL) in the cerebrospinal fluid (CSF).

TEST	ELEVATED NFL(B)N = 12Mean (range) SD	NORMAL NFL(B)N = 14Mean (range) SD	P-valueElevated vs. normal
**ROCF** 1			
Copy (max 36)	32.1 (8–36) 9.1	35.5 (4–32) 1.1	0.80
Delay (max 36)	18.0 (3.5–31) 9.5	171 (1–31) 7.7	0.67
**VOCABULARY** 2 **(max 70)**	28.4 (16–47) 11.5	34.3 (17–60) 11.4	0.16
**COWAT** 3	32.2 (18–48) 11.2	39.3 (19–54) 11.4	0.09
**DIGIT SPAN** 4 **(max 14)**	6.8 (4–10) 2.1	7.3 (3–11) 2.0	0.40
**LISTENING SPAN** 5 **(max 38)**	12.8 (1–21) 6.1	15.4 (6–33) 8.3	0.56
**TRAIL MAKING** 6			
Part A, (s)	35.8 (20–75) 15.7	26.2 (15–55) 9.9	**0.04**
Part B, (s)	98.7 (43–240) 54.7	69.0 (45–100) 18.1	0.18
**REACTION TIME** 7			
Simple (msec)	371.1 (259.6–956.8) 191.3	285.1 (242.0–361.6)	**0.04**
Difference (complex-simple, msec)	126.2 (−248.0–388.0) 161.7	349.6 (92.7–3087.9) 733.3	0.84
**FINGER TAPPING** 8			
Dominant Hand	48.3 (36.6–67.2) 9.1	45.4 (29.1–61.3) 11.5	0.59
Non-Dominant Hand	55.7 (41.2–70.2) 9.4	49.2 (31.3–70.4) 11.4	0.17
**EPISODIC MEMORY** 9			
Auditory – Remember HR	0.42 (0.05–0.95) 0.32	0.50 (0.14–0.77) 0.20	0.34
Auditory – Know HR	0.26 (0.05–0.86) 0.29	0.20 (0.05–0.59) 0.14	0.96
Visual – Remember HR	0.50 (0.05–0.86) 0.28	0.43 (0.05–0.86) 0.19	0.52
Visual – Know HR	0.17 (0.05–0.41) 0.14	0.24 (0.05–0.68) 0.19	0.39

NFL in the cerebrospinal fluid has been analyzed in the same cohort of boxers and the results are previously published [9]. After a boxing bout, cerebrospinal fluid was collected at two occasions, first 1–6 days (test A) after bout and then after a rest period of at least 14 days (test B). 23 of 30 boxers had elevated concentrations of the axonal injury biomarker NFL at test A. At follow up, 4 of the boxers were lost, 12 of 26 (46%) boxers still hade elevated concentrations and these had significantly lesser performance on Trail Making A (p = 0.041) and Simple Reaction Time (p = 0.042) compared to the other boxers.

1 *ROCF: Rey Osterrieth Complex Figure Test.*

2 *Vocabulary: 35 words were presented and for each word 0,1 or 2 points were assigned.*

3 *COWAT (Controlled Word Association Test): Participants were asked to generate as many words as possible that begin with the given letters F, A and S. Sixty seconds were allowed for each letter. One point per word was received.*

4 *Digit Span: Participants were presented with series of numbers, starting with three (for example 3–4–8). There were two series in each level and in total seven levels. The task was to immediately repeat the numbers back. When doing this successfully, the participants were given a longer serie of numbers.*

5 *Listening Span: A mixed lists of digits and letters were read aloud to the participants and they were asked to recall this list in correct numeric and alphabetic order.*

6 *Trail Making: In trail A the participant is instructed to trace a line that connects circled numbers in consecutive order. In trail B the task is to trace a line by alternating circled numbers and circled letters in consecutive order. Time is measured in seconds(s).*

7 *Reaction time: a) Simple reaction time: Participants were instructed to respond as quickly as possible whenever the circle appeared on the screen. Complex reaction time: Required participants to respond with right hand to the circle and left hand to the triangle. Difference = Complex−Simple reaction time.*

8 *Finger Tapping. In total five trials with 15 s rests between the trials. The participants were asked to press the space board with their index finger on the computer keyboard as many times as possible for 15 s, alternating dominate and non-dominant hand. The mean value was calculated.*

9 *Proportional values (range 0.04–0.96). The number of words in the auditory and visual conditions was 30 respectively. The correct recognition hit rates (HR) are presented.*

Differences in the neuropsychological test results were seen between boxers with abnormal, as compared to normal, CSF NFL at test B (follow-up sample after rest from boxing). Boxers with prolonged NFL elevation had deficits regarding Trail Making A (p = 0.041) and simple reaction time (p = 0.042) (table 2). Comparisons for NFL in closer relation to bout (test A) revealed no statistical significances.

## Discussion

None of the neuropsychological assessment tools could reveal any impairment in the boxers, when compared to controls, although our test battery included attention, processing speed, working memory and visual learning; all the areas where impairment after mild TBI has been previously demonstrated [59]. The performance in some of the boxers was very poor, but similar results were also found among controls. In most tasks the performance was identical or slightly in favor for the boxers.

We have previously in the same cohort of boxers been able to show that the repetitive subconcussive trauma in amateur boxing causes axonal injury detectable by CSF biomarkers [9], [11]. This is the first study to investigate the relationship between neuropsychological evaluation and CSF NFL (an axonal injury biomarker [9], [12]), and the results were quite interesting.

Amateur boxers still having increased CSF NFL concentrations after a rest period of at least 14 days, had significantly worse performance on two (out of four) tasks involving processing speed responses, Trail Making part A (p = 0.04) and Simple Reaction Time (p = 0.04), compared to the other boxers. The boxers with prolonged elevation of CSF NFL had most likely received more punches during bout leading to greater degree of axonal injury, compared to the other boxers [9], [12]. Another interpretation is that these boxers may suffer from ongoing neurodegeneration. This interpretation may be speculative but in our mind not irrelevant in the light of recent data on progressive neurodegeneration following repeated concussions [60]. The association of CSF NFL with speed measures gives a functional correlate to the CSF biomarker findings and calls for further investigations with repeated CSF samplings and long-term clinical and neuropsychological follow-up within this group.

Our study has some limitations: First, the absence of baseline neuropsychological assessment before bouts may make it hard to determine if the test scores reflect the actual boxing bout. Second, since several statistical tests were performed, there is a risk of type I errors, why the observed relationship between processing speed and prolonged increase in CSF NFL concentration need to be repeated in independent studies before stronger conclusions can be drawn. Whether baseline testing has greater diagnostic accuracy than post concussion assessment alone still remains to be determined [61], since also the test-retest reliability must be taken into account [62] and findings further suggest that post injury neuropsychological test data are robust and may not require baseline testing, as long as there exist appropriate, well-developed normative data [63]. However, traditional test norms are based upon quota sampling, the recruitment of a normative sample reflecting the demographic properties of the national census. A problem in investigations involving clinical patients, ethnical minorities, or groups with special skills (such as the athletes in our study) is that these populations often do not reflect the national census in crucial aspects. Hence, census-based norms may bias the interpretation of results in unpredictable ways. In our study, such a method would have overestimated the effects of boxing upon language and possibly episodic memory. Over the years, several different methods for correcting recruitment bias have been suggested [64]. We tried to cope with this problem by recruiting a group of scrupulously matched controls. It should be noted, that this approach in the future should be supplemented with norms derived from meta-analyses of boxer studies. Using Monte Carlo simulation, census-based norms can then be adjusted to reflect recruitment bias. Such a necessary enterprise requires the publication of a larger number of studies than currently available.

The clinical value and validity of neuropsychological assessment in the diagnosis and monitoring of mild TBI has been under discussion. Until now, neuropsychological evaluation has been advocated as the most sensitive tool for detecting early neurological pathology, but our results indicate that without baseline testing and in absence of clinical symptoms, neuropsychological assessment cannot detect the small axonal injuries caused by the repetitive subconcussive trauma in boxing. However, specific test of processing speed and executive functions may indicate protracted or delayed injury and may hold promise as markers for more severe trauma. The clinical relevance of elevated CSF NFL without clinical symptoms or without cognitive impairment can be discussed, but earlier studies have shown that concussion increases the risk for additional concussions [51], that at least 12% of the athletes with fatal sport-related intracerebral bleeding have suffered from a concussion within 4 weeks prior to the injury [65] and that sport-related concussion is associated with long-term effects in form of chronic traumatic encephalopathy [60]. The results of these studies indicate that the risk for complications increases if athletes return to sport before the traumatic brain injury has healed and that it today is difficult to decide correctly when that is the case. Just as in fracture healing, absence of clinical symptoms might not be equivalent with absence of/healed traumatic brain injury, why more sensitive analysis tools, such as analysis of CSF brain injury biomarkers, might be helpful for the clinician.

In the future, it would be valuable also investigate the relationship between CSF NFL and some of the established neuropsychological computerized concussion batteries of today (Immediate Postconcussion Assessment and Cognitive Testing (ImPACT), Axon Sports, the Automated Neuropsychological Assessment Metrics (ANAM), Headminder (ImPACT Applications, Inc; Axon Sports, LLC)) in concussed athletes.

### Conclusion

This is to our knowledge the first study showing traumatic axonal brain injury can be present without measureable cognitive impairment. Absence of clinical symptoms/cognitive impairment after concussion does not seem to be equivalent to absence of brain injury. Our conclusion is that assessment of memory tests, tests of processing speed or executive functions, cannot detect the small axonal injuries that can be diagnosed with CSF NFL analysis in amateur boxing. However, these modalities can provide complementary information and link CSF biomarker results to functional outcome.
